# Dose distribution near thin titanium plate for skull fixation irradiated by a 4-MV photon beam

**DOI:** 10.4103/0971-6203.62199

**Published:** 2010

**Authors:** Tomohiro Shimozato, Keisuke Yasui, Ryota Kawanami, Kousaku Habara, Yuichi Aoyama, Katsuyoshi Tabushi, Yasunori Obata

**Affiliations:** Department of Radiological Technology, Nagoya University School of Health Sciences, Higashi-ku, Nagoya, Aichi, Japan; 1 Nagoya University Graduate School of Medicine, Higashi-ku, Nagoya, Aichi, Japan; 2 Department of Radiotherapy, Nagoya University Hospital, Showa-Ku, Nagoya, Aichi, Japan

**Keywords:** Monte Carlo simulation, photon beam, radiation treatment planning system, scattered radiation, titanium plate

## Abstract

To investigate the effects of scattered radiation when a thin titanium plate (thickness, 0.05 cm) used for skull fixation in cerebral nerve surgery is irradiated by a 4-MV photon beam. We investigated the dose distribution of radiation inside a phantom that simulates a human head fitted with a thin titanium plate used for post-surgery skull fixation and compared the distribution data measured using detectors, obtained by Monte Carlo (MC) simulations, and calculated using a radiation treatment planning system (TPS). Simulations were shown to accurately represent measured values. The effects of scattered radiation produced by high-Z materials such as titanium are not sufficiently considered currently in TPS dose calculations. Our comparisons show that the dose distribution is affected by scattered radiation around a thin high-Z material. The depth dose is measured and calculated along the central beam axis inside a water phantom with thin titanium plates at various depths. The maximum relative differences between simulation and TPS results on the entrance and exit sides of the plate were 23.1% and – 12.7%, respectively. However, the depth doses do not change in regions deeper than the plate in water. Although titanium is a high-Z material, if the titanium plate used for skull fixation in cerebral nerve surgery is thin, there is a slight change in the dose distribution in regions away from the plate. In addition, we investigated the effects of variation of photon energies, sizes of radiation field and thickness of the plate. When the target to be irradiated is far from the thin titanium plate, the dose differs little from what it would be in the absence of a plate, though the dose escalation existed in front of the metal plate.

## Introduction

For treatment of brain tumors by cerebral nerve surgery, external radiotherapy is carried out after surgery to remove the surviving tumor or prevent its recurrence. During surgery, the skull is cut open, the tumor removed and the opened skull refixed using a high-Z–material plate.[1] When the plate is embedded in the skull and the skull/ brain is irradiated, the dose distributions can be affected by the radiation scattered by the plate.[2]

A radiation treatment planning system (TPS) is used in external radiotherapy to calculate how radiation doses are distributed in, and absorbed by, the body. TPS dose calculations assume dose distributions as measured in water and do not address changes that might accrue due to radiation scattering in high-Z materials. However, since it is difficult to irradiate the tumor while avoiding the metal plate, the tumor and its surroundings, including the plate, are irradiated during external radiotherapy. An image obtained by computed tomography (CT) shows a radial artifact around a high-Z material, suggesting that the relative electron density required for calculating the dose absorbed in the body by the TPS cannot be used to accurately convert the CT number using the pixel-by-pixel method. Thus, methods that calculate dose distributions in internal organs or in high-Z materials as if they were water are of questionable value. Indeed, a previous study reports that dose distributions calculated using a water-equivalent phantom differ from those measured in water that contains metallic materials.[3] Other relevant studies clarify the effects of scattered radiation around high-Z materials used for hip prosthesis[3–9] and dental implants[10–14] and the effects on tissue around a thick metal.

We investigated the distributions of a radiation dose that scatters when a thin titanium plate (thickness, 0.05 cm) used for skull fixation in cerebral nerve surgery (as other researchers could not perform) is irradiated. First, because it is difficult to measure radiation using an ionization chamber when a titanium plate is embedded in the skull, we performed measurements by irradiating film placed vertically against the central beam axis of a water-equivalent phantom with a thin titanium plate at 5-cm depth, similar to the experimental geometry used in the study by Farahani *et al.*[11] Next, using a computational phantom simulating water with a bone of a human head simulated as being between two slices of titanium plate, we calculated radiation dose distributions along a transverse section of the phantom by Monte Carlo (MC) simulation and TPS. We compared the measured and calculated results to determine how dose distribution is affected by scattered radiation and how two-dimensional dose distributions calculated by simulation differ from those calculated by TPS.

## Materials and Methods

**Figure 1:**
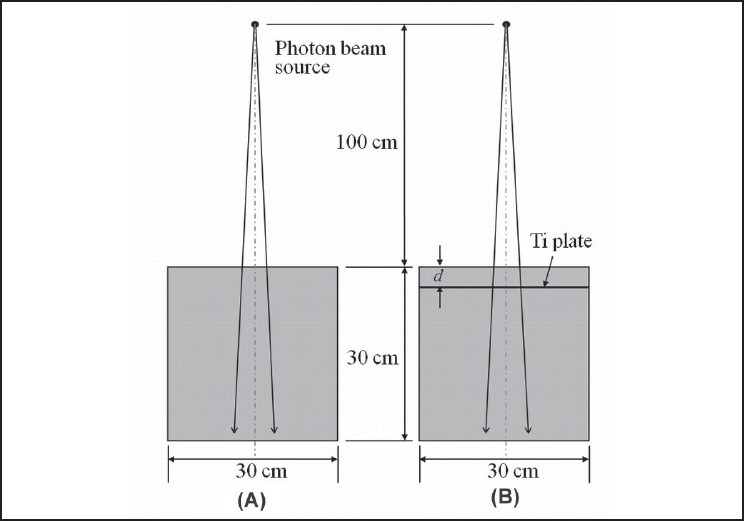
Experimental setup and simulation geometry for acquisition of depth dose data in water: (A) without titanium plate and (B) with titanium plate. *d* is the distance from the surface of the water phantom to the surface of the plate on the entrance side

Measurements were performed using a medical linear accelerator (Varian Clinac 21EX, Varian Medical Systems, Palo Alto, CA). Specifications for all measurements, simulations and calculations were as follows: field = 5 × 5 cm^2^; constant source-to-surface distance (SSD) = 100 cm; 4-MV x-ray beam. Tissue-phantom ratio (TPR) at depths of 20 cm divided by TPR at depth of 10 cm was 0.616 (TPR_20,10_). The experimental setup and simulation geometry, with and without a titanium plate, are shown in Figure 1.

MC simulations were performed using MC code (Electron Gamma Shower, version 5; EGS5[15]). TPS calculations were performed using a commercial TPS (XiO version 4.33.02, CMS Inc., St. Louis, MO) which was commissioned for the medical linear accelerator used in the measurements.

### Measurement of depth dose in water phantom

Depth doses in water were measured using a farmer-type ionization chamber (TM30013, PTW-Freiburg; volume, 0.6 cm^3^) placed along the central beam axis of a photon beam. Measurements in simple experimental geometry, as shown in Figure 1A, were carried out in a water tank (MP3, PTW-Freiburg) with software (Mephysto software, version 7.42) for therapy beam analysis. Measurements were taken from the surface of the water to 30-cm depth using an electrometer (PTW-Unidos, PTW-Freiburg).

### Simulation by EGS5 of depth dose data in water phantom

Depth dose data were obtained by EGS5 simulation using a computational phantom simulating water (size, 30 × 30 × 30 cm^3^) irradiated by a photon beam (as described above). Data were acquired from the surface to a depth of 30 cm along the central beam axis. The voxel size for calculation was 0.5 × 0.5 × 0.05 cm^3^. Statistical uncertainties for depths up to 30 cm in water were less than ±1%. Statistical uncertainties were calculated by the method.[16] This method calculates the fractional standard deviation (FSD) as the statistical error by means of the standard variance of mean divided by mean value. Cutoff energies for electrons (ECUT) and photons (PCUT) were set to 0.521 and 0.01 MeV, respectively. The 4-MV spectrum of the Varian linear accelerator, calculated by Sheikh-Bagheri and Rogers,[17] was used. Dose distributions were calculated from the EGS5 simulation results by changing energy per unit volume (J/m^3^) into energy per unit mass (J/kg); that is, the energy deposited per unit volume was divided by the density of water (1000 kg/m^3^).

### Calculation by TPS of depth dose in water phantom

Depth doses were calculated by XiO TPS along the central beam axis using a computational phantom simulating water, similar to the one described above. The voxel size in the dose calculation grid was set to 0.05 cm. Dose calculations were performed using the superposition algorithm. A photon beam was irradiated vertically against the surface of the phantom, and the depth dose along the central beam axis in computational phantom simulating water was calculated. The relative electron density was normalized to the electron density of water [Table 1].

**Table 1:** Atomic number, physical density and relative electron density of studied materials

*Element*	*Water[22]*	*Tough water phantom[23]*	*ICRU Bone[22]*	*Titanium[24]*
Effective atomic number (*Z_eff_*)^a^[21]	7.51	8.00	12.94	22.00
Physical density (g/cm^3^)	1.000	1.017	1.610	4.540
Electron density (electron/cm^3^)	3.343 × 10^23^	3.311 × 10^23^	5.071 × 10^23^	1.257 × 10^24^
Relative electron density of water	1.000	0.989	1.517	3.759

a Z_eff_ = (∑*_i_w_i_Z_i_*^3.5^)^1/3.5^, where *w_i_* is the fraction by weight of element *i* having atomic number *Z_i_*

### Measurement of depth dose in the presence of a titanium plate

A thin titanium plate (thickness, 0.05 cm) was embedded in a tough water phantom (WE-211, Kyoto Kagaku Co.) at various depths *d* as shown in Figure 1B. Depth doses were then measured, obtained by simulation, and calculated by TPS.

Depth doses were measured in the presence of the titanium plate at *d* = 5 cm in the above phantom. Physical specifications are shown in Table 1. The size of the tough water phantom was 30 × 30 cm^2^, and the phantom was of the slab type. First, for a calibration curve, optical density was measured by changing the exposure dose for radiochromic film (Gafchromic EBT, ISP. Inc.) placed 10 cm below the surface of the tough water phantom. Next, the depths of the films fixed perpendicular to the central beam axis were 2.00, 5.00 (incident side of the plate), 5.10 (exit side of the plate), 10.15, 15.15 and 20.15 cm from the surface of the phantom with the titanium plate at depth of 5 cm. The tough water phantom, with titanium and film embedded, was irradiated by the 4-MV photon beam. Post-irradiation film growth was about 9% to 11% within 6 hours and negligible after more than 10 hours,[18] so the film was scanned after a lapse of more than 10 hours after irradiation, in this study. EBT film was selected because the atomic number and density of the film are close to those of water. The effective atomic number and density of EBT film are 7.05 and 1.1 g/cm^3^, respectively. Then, near-energy independence in the range of photon energies from 30 keV to 30 MeV has been reported.[19] The film was scanned using an ES-10000G flat-bed scanner (EPSON Co.) equipped with a broad-spectrum light source. The resolution for scanning was 150 dpi. Then, the films were read using film-analysis software (DD-system, version 9.0.0.0, R-TECH Inc.).

### Simulation by EGS5 of depth dose in the presence of a titanium plate

The titanium plate was embedded at various depths *d* = 0.5, 1.0, 2.0 and 5.0 cm along the central beam axis from the surface of the computational phantom simulating water. The depth dose from the surface of the computational phantom to the incident side surface of the plate was obtained for comparison with the depth dose data obtained using XiO. Simulation parameters (histories, PCUT, ECUT, voxel size for calculation and so on) were as described above. The 4-MV spectrum calculated by Sheikh-Bagheri and Rogers[17] was used, as mentioned previously. The physical densities of water and titanium are shown in Table 1.

The following dependences were investigated by EGS5 simulation using backscattered dose perturbation factors (BSDFs) and forward dose perturbation factors (FDPFs) of the ratio of doses with and without the presence of the interface indicated in AAPM (The American Association of Physics in Medicine) TG-63.[3] We calculated the BSDFs and FDPFs for variations of depth of plate (0.5, 1, 2 and 5 cm), of field size of photon beam (3 × 3, 5 × 5 and 10 × 10 cm^2^), of photon energy (4, 6 and 10 MV) and of plate thickness (0.05, 0.1, 0.5, 0.7 and 1.0 cm).

### Calculation by TPS of depth dose in the presence of a titanium plate

Depth dose was calculated by TPS using the superposition algorithm with the same geometrical setup and plate depths as for the simulation. The TPS requires relative electron density of water for the evaluation of radiation interaction with the titanium plate, which was obtained by the following equation.[4,20]

(1) $$\rho_{e}=\rho \cdot N_{A}\cdot \sum \frac{w_{i}\cdot Z_{i}}{A_{i}}\;\left(electron/cm^{3}\right)$$

where *N_A_* is Avogadro's number (6.022045 × 10^23^/mol), ρ is the physical density, Z_*i*_ and A_*i*_ are the atomic number and atomic weight, respectively, of the *i*^th^ element and *w*_i_ is its proportion by weight. Relative electron densities were calculated by dividing the electron densities of the material by the density of water. The effective atomic number, physical density and relative electron density of titanium, water, ICRU (International Commission on Radiation Units& Measurements) bone and tough water phantom are listed in Table 1 [calculated from equation (1)].[21–24] The depth dose was calculated along the central beam axis in computational phantom simulating water.

### Dose distribution data for a simulated clinical case

**Figure 2:**
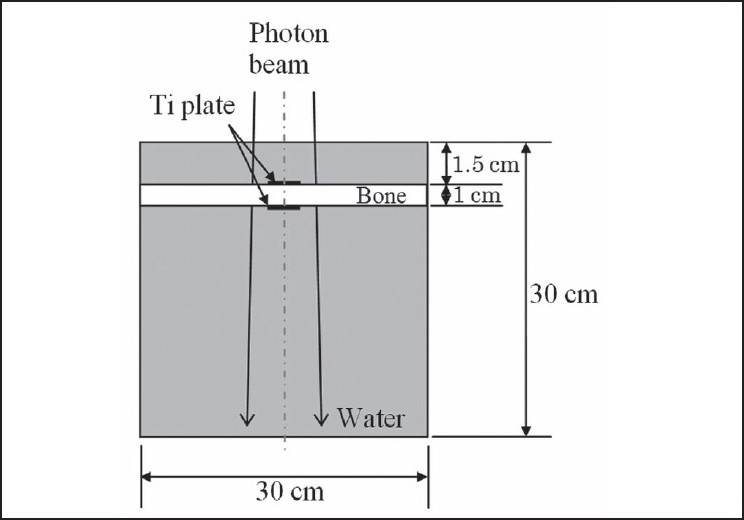
Geometry of computational water-equivalent phantom with skull simulated as being between two slices of titanium plate. The field size is 5 × 5 cm^2^ at the isocenter

Dose distribution data were also obtained for a computational phantom simulating water with a 1-cm–thick bone (ICRU Report 46, skeleton-cranium) simulated as being between two slices of 0.05-cm–thick titanium plate (2 × 2-cm^2^ square), as shown in Figure 2. The thickness, shape, width and length of the metal plate were set to those of a titanium plate manufactured by Bioplate, Inc.

Two-dimensional dose distributions were calculated by EGS5 simulation. The voxel size for calculation was 0.1 × 0.1 × 0.05 cm^3^ to evaluate scattered radiation to lateral side in detail. Statistical uncertainties of less than ±1% were achieved in the primary beam. Physical density is given in Table 1. The parameters of the simulation (histories, PCUT, ECUT, voxel size for calculation and so on) were as described above.

Two-dimensional dose distributions were also calculated by TPS. The relative electron density is given in Table 1. The voxel size in the calculation grid was set to 0.05 cm.

## Results and Discussion

### Depth dose data

**Figure 3:**
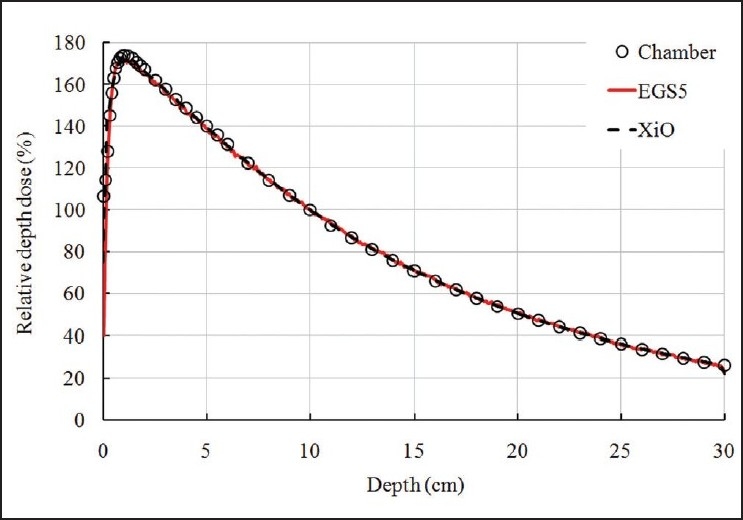
Depth doses along the central axis inside a water phantom that are irradiated by a 4-MV photon beam with a 5 × 5-cm^2^ field at the isocenter. Data were obtained using the ionization chamber by measurement (circles), EGS 5 simulation (solid red line) and XiO TPS (dashed black line)

Depth dose curves were measured in water along the central beam axis in an ionization chamber. Data calculated by TPS were fitted with a precision of ±1%. Data obtained by EGS5 simulation and calculated by TPS differed by 0.25% ± 0.86% (average ± standard deviation). Depth dose curves are shown in Figure 3. Each value in the data was normalized to the dose value at *d* = 10 cm from the surface of the phantom. The EGS5 simulation results, thus, were in agreement with TPS results and with the measured values.

### Depth dose data in the presence of a titanium plate

**Figure 4:**
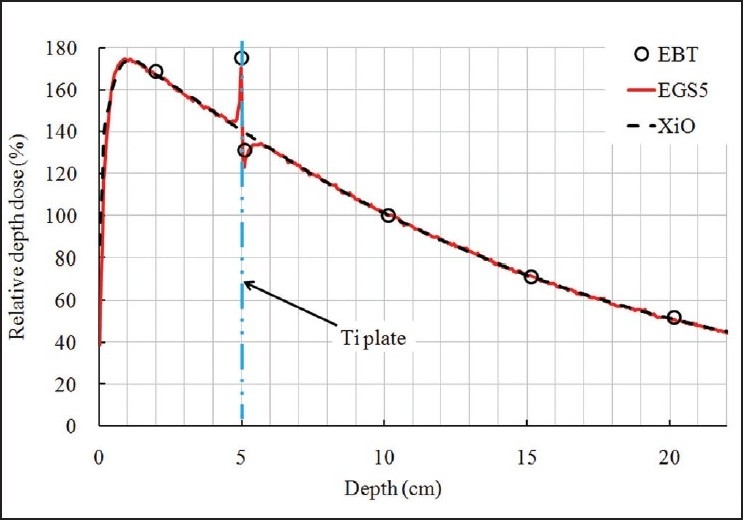
Depth doses along the central axis inside a water phantom fitted with a titanium plate (thickness, 0.05 cm) at 5-cm depth. Data were obtained by measurement (circles), EGS5 simulation (solid red line) and XiO TPS (dashed black line) for a 4-MV photon beam

In the presence of a thin titanium plate, depth dose values were measured by EBT film, calculated by XiO TPS and obtained by EGS5 simulation. Results are shown in Figure 4. The ratios of simulation values to measured values were 0.989, 0.965, 0.946, 1.000, 1.000 and 0.988 at *d* = 2.00, 5.00 (entrance side of the plate), 5.10 (exit side of the plate), 10.15, 15.15 and 20.15 cm, respectively, with the plate at a depth of 5 cm. Data were normalized at *d* = 10.15 cm. EGS5 simulation values agree with measured values and are thus confirmed to be suitable for evaluating perturbations in dose distribution in the presence of a plate. Dose calculation uncertainties were found to be the greatest near the plate, due perhaps to voxel size. Measured values and simulation values immediately in front of and behind the plate differ greatly from the TPS values, presumably because the superposition algorithm used in TPS calculation does not reliably consider both atomic number and variation in density, as proved by Miften *et al.*[25]

**Figure 5:**
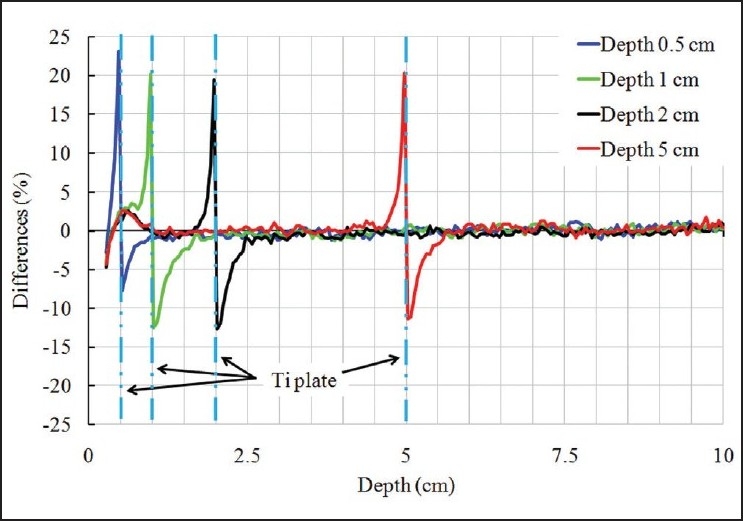
Comparison of depth doses calculated by MC (EGS5) simulation and XiO TPS

The relative differences between simulation and TPS results in the presence of the plate at various depths are shown in Figure 5. The maximum relative difference in backscattered radiation on the entrance side of the plate between simulation values and TPS-calculated values was 23.1%. Similarly, the maximum relative difference in front-scattered radiation on the exit side of the plate was – 12.7%. Each value in the data was normalized to the dose value at *d* = 10 cm. Thus, the TPS calculation using the superposition algorithm underestimates the dose at the entrance side of the plate and overestimates the dose on the exit side of the plate.

For variations in depth, BSDFs were found to be 1.208, 1.206, 1.198 and 1.206, and FDPFs were found to be 0.921, 0.892, 0.881 and 0.894 for depths of 0.5, 1, 2 and 5 cm, respectively. BSDF and FDPF are essentially independent of plate depth. Variation in depth due to scalp thickness might not affect dose distributions around the plate. For variation in field size, BSDFs were found to be 1.218, 1.206 and 1.189, and FDPFs were found to be 0.904, 0.894 and 0.889 for fields of 3 × 3, 5 × 5 and 10 × 10 cm^2^, respectively. These factors decrease slightly as field size increases. Moreover, Das and Khan[2] reported that the energy dependence was little for the energy region of radiation from 60Co (mean 1.25 MeV) to 10 MV except for lead. For variations in energy, BSDFs were found to be 1.206, 1.195 and 1.182, and FDPFs were found to be 0.894, 0.939 and 0.990 for beams of 4, 6 and 10 MV, respectively. The dependence on energy of a radiotherapy photon beam is small. For variation in plate thickness, as calculated by our additional simulation, BSDFs were found to be 1.192, 1.200, 1.208, 1.216 and 1.223, and FDPFs were found to be 0.884, 0.848, 0.819, 0.801 and 0.783, for thicknesses of 0.05, 0.1, 0.5, 0.7 and 1 cm, respectively. BSDF increases for thickness up to 1 cm, and FDPF decreases with increasing thickness. It is possible that the thickness changes by means of incident angle of photon beam, though we calculated for the thickness of plate in common clinical use. Thus, it is possible that depth dose immediately in front of or behind the plate might increase or decrease due to factors such as field size, photon energy and plate thickness. It is also possible that plate materials with atomic numbers higher than the atomic number of titanium might cause more scattering, as reported by Das and Khan.[2]

**Figure 6:**
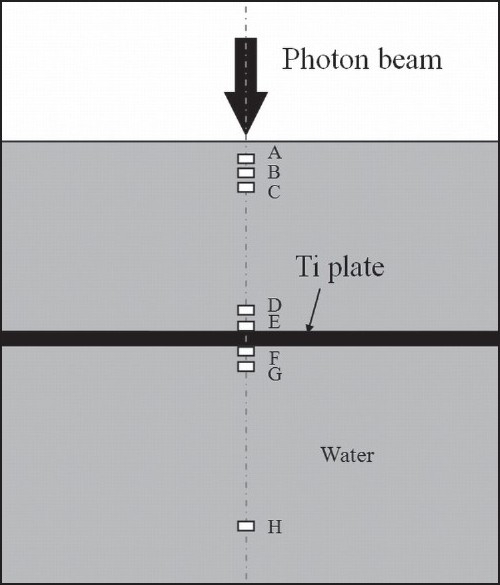
Calculation region for analyzing radiation absorbed at various regions when a titanium plate (thickness, 0.05 cm) is embedded in a computational water-equivalent phantom. Distances from the phantom surface, for calculation, are as follows: (A) 0.075 cm, (B) 0.225 cm, (C) 0.375 cm, (D) 4.825 cm, (E) 4.975 cm, (F) 5.075 cm, (G) 5.225 cm and (H) 10.025 cm

We investigated the effect of the plate on radiation scattering by studying the post-irradiation deposition of three different secondary products (photon, electron and positron) for the computational phantom simulating water with and without a plate of high-Z material (*d* = 5 cm). Calculation voxels were centered at depths of 0.075, 0.225, 0.375, 4.825, 4.975, 5.075, 5.225 and 10.025 cm from the phantom surface, respectively [Figure 6]. Table 2 shows the values of deposited dose with plate divided by dose without plate in each of the calculation regions: buildup region, entrance side of the plate and exit side of the plate. Dose varies with the number of secondary electrons: when secondary electrons on the entrance side of the plate bounce from the plate, dose increases; when they are absorbed by the plate on the exit side of the plate, dose decreases. Dose does not vary with the number of photons.

**Table 2:** Ratio of radiation dose deposited in water with plate to that deposited in water without plate

*Calculation region*		*Total*	*Photon*	*Electron*	*Positron*
Buildup region	A	1.001	0.936	1.001	1.037
		(1.001)	(1.000)	(1.004)	(1.026)
	B	1.005	0.979	1.004	1.099
		(1.001)	(1.000)	(1.003)	(1.088)
	C	0.998	1.131	0.998	0.988
		(0.998)	(1.001)	(0.995)	(0.951)
Entrance side of plate	D	1.025	0.990	1.025	1.247
		(1.012)	(1.001)	(1.024)	(1.229)
	E	1.192	0.859	1.191	1.381
		(1.086)	(1.002)	(1.189)	(1.446)
Exit side of plate	F	0.885	0.626	0.881	2.093
		(0.924)	(0.998)	(0.831)	(1.823)
	G	0.951	0.762	0.949	1.531
		(0.979)	(0.997)	(0.956)	(1.482)
10.025 cm depth from surface	H	0.993	1.125	0.993	0.942
		(0.992)	(0.994)	(0.991)	(0.953)

Values in parentheses are the relative numbers of radiation particles that arrive in, and are generated at, the calculation regions for water without plate.

Differences between simulation and TPS results are large near the plate. However, the presence of a thin plate in the human body does not significantly affect the dose given to a brain tumor far from the plate because the variance in dose at *d* = 10.025 cm is only 0.993 for water without plate, as shown in Table 2. A difference of up to ±1% is virtually insignificant in clinical treatment. It is necessary to control so as to not exceed tolerance dose of normal tissue, due to the presence of the plate.

### Dose distribution data for a simulated clinical case

**Figure 7:**
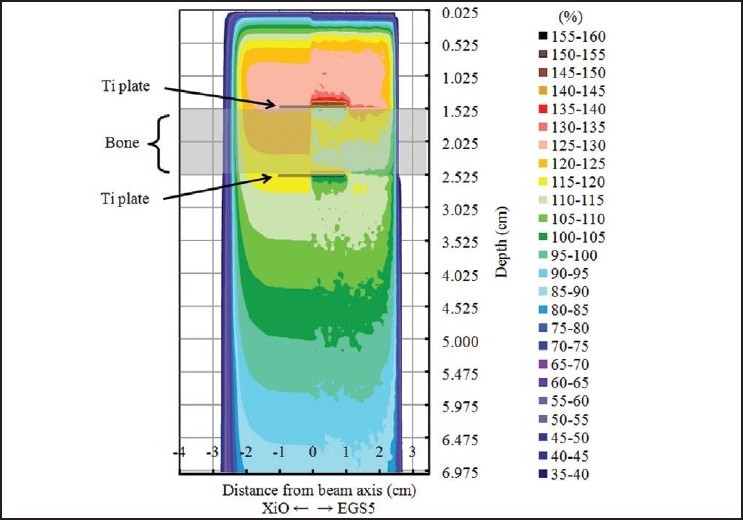
Two-dimensional distributions in a clinical situation. Left:distribution calculated by XiO TPS using the superposition algorithm. Right: distribution calculated by MC (EGS5) simulation. The ICRU bone sandwiched between two titanium plates (each of thickness 0.05 cm) is at a depth of 1.5-2.5 cm from the surface of the computational phantom simulating water

Two-dimensional dose distributions when a skull was fitted with a thin titanium plate as in a clinical case were obtained. The left side of Figure 7 shows the results calculated by XiO TPS using the superposition algorithm, while the right side shows the results obtained by EGS5 simulation. All data were normalized to the dose value at *d* = 5 cm along the central beam axis.

TPS results, with the exception of the buildup region and around the titanium plate or bone, agree with simulation results, although the latter show a characteristic perturbation of MC simulation. TPS calculations using the superposition algorithm do not accurately consider the effects of radiation scattering due to the presence of high-Z material. In contrast, the simulation shows the effects of not only backscattered and forward perturbation but also lateral scattered radiation. When radiation enters a high-Z material, the depth dose increases in front of the plate due to backscattered radiation caused by bouncing of photons and secondary electrons. The lateral dose at the plate also increases, due to lateral scattering from the plate and backscattering from the bone. A cold spot is generated on the exit side of the plate by electron absorption and a decrease in photons inside the plate as described above. Buildup on exit side of plate increases again when photons pass from higher-density to lower-density material.

Differences between simulation and TPS results in the depth and lateral directions are large at distances less than 1 cm from the plate and small at distances greater than 1 cm. Therefore, the dose distribution around a tumor that is more than 1 cm from the plate is the same as that calculated by TPS. However, a treatment planner who uses a TPS to plan doses should set the irradiation angle and field so as to avoid the plate as much as possible, especially if the plate material is unknown. In particular, for high-Z materials, it is important to consider the effect of scattered radiation when calculating doses using any brand of TPS, except for one that uses the MC algorithm.

## Conclusions

Thin titanium plates used for skull fixation in cerebral nerve surgery affect dose calculations for post-surgery radiation therapy. When the target to be irradiated is far from the titanium plate, the dose differs little from what it would be in the absence of the plate. However, when the target to be irradiated is near the plate, doses calculated by TPS using the superposition algorithm do not accurately consider radiation scattering around the plate and thus might differ from actual received doses.

In this study, the basic data of the effect of scattered radiation due to a metal plate for 4-MV photon beam were obtained in water phantom embedded with thin titanium plate. We hope that our results are applicable as reference in clinical treatment.
